# Acute Flaccid Myelitis: Something Old and Something New

**DOI:** 10.1128/mBio.00521-19

**Published:** 2019-04-02

**Authors:** David M. Morens, Gregory K. Folkers, Anthony S. Fauci

**Affiliations:** a National Institute of Allergy and Infectious Diseases, National Institutes of Health, Bethesda, Maryland, USA; Johns Hopkins Bloomberg School of Public Health

**Keywords:** acute flaccid myelitis, emerging diseases, enterovirus, neurology

## Abstract

Since 2014, acute flaccid myelitis (AFM), a long-recognized condition associated with polioviruses, nonpolio enteroviruses, and various other viral and nonviral causes, has been reemerging globally in epidemic form. This unanticipated reemergence is ironic, given that polioviruses, once the major causes of AFM, are now at the very threshold of global eradication and cannot therefore explain any aspect of AFM reemergence. Instead, the new AFM epidemic has been temporally associated with reemergences of nonpolio enteroviruses such as EV-D68, until recently thought to be an obscure virus of extremely low endemicity. This perspective reviews the enigmatic epidemiologic, virologic, and diagnostic aspects of epidemic AFM reemergence; examines current options for clinical management; discusses future research needs; and suggests that the AFM epidemic offers important clues to mechanisms of viral disease emergence.

## Abstract (precis)

Since 2014, acute flaccid myelitis (AFM), a long-recognized condition associated with polioviruses, nonpolio enteroviruses, and various other viral and nonviral causes, has been reemerging globally in epidemic form. This unanticipated reemergence is ironic, given that polioviruses, once the major causes of AFM, are now at the very threshold of global eradication and cannot therefore explain any aspect of AFM reemergence.

## PERSPECTIVE

**FIG 1:**
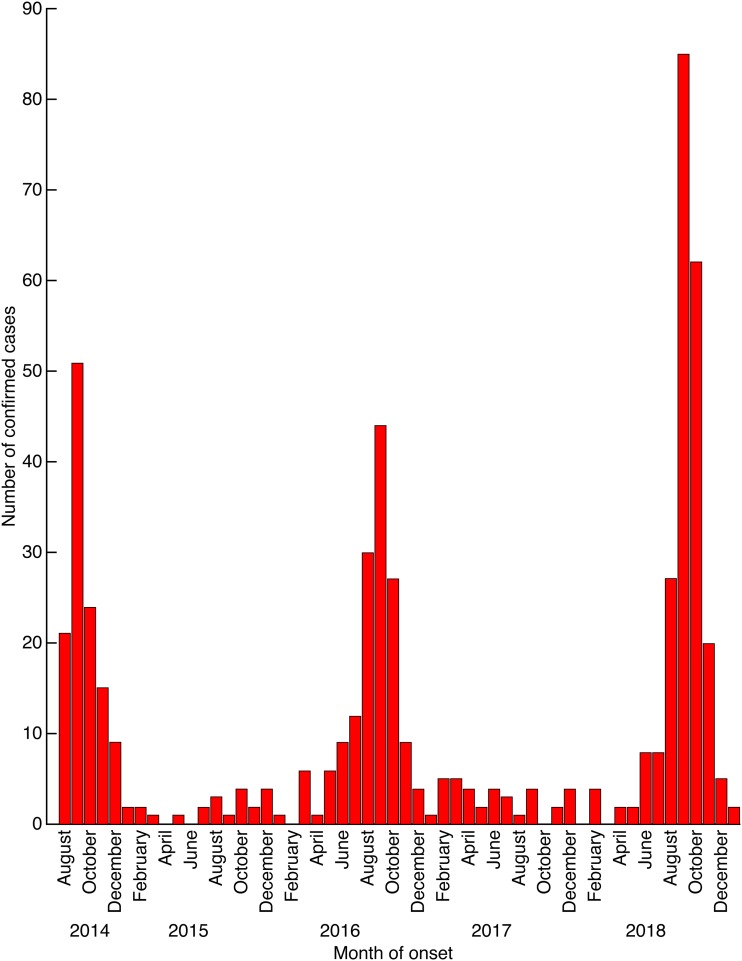
Epidemic curve of 551 confirmed cases of AFM reported to the U.S. Centers for Disease Control and Prevention by month of onset, 14 August 2014 to 31 January 2019 (6). The epidemic curves of summer/fall AFM correspond closely to typical seasonal peaks of most NPEVs, including EV-D68 (7, 8). Note that the U.S. epidemics have occurred in 2-year cycles, with peak case onsets in the middle week of September in 2014, 2016, and 2018 but with few cases during any season of the intervening years 2015 and 2017.

In recent decades, new human infectious diseases such as HIV/AIDS, severe acute respiratory syndrome (SARS), and Nipah virus infection, among others have emerged. Well-known diseases also have reemerged because of human movement, crowding, and other population factors (e.g., dengue and dengue hemorrhagic fever), warfare and natural disasters (e.g., cholera), and viral evolution (e.g., poultry-adapted influenza A H5N1 and H7N9) (1). Joining this list is epidemic acute flaccid myelitis (AFM), characterized by sudden denervation-associated muscle paralysis of healthy children (and occasionally adults) in one or more limbs that mimics poliomyelitis but which is not caused by polioviruses (2). AFM was first recognized around 2010 as a seemingly novel condition (3, 4) and quickly grew into an alarming and important disease threat, with the first large outbreak occurring in 2014 (5). Since then, seasonal waves have occurred every other year in the United States, the largest occurring in 2018 (Fig. 1) (6–8)). Because of its uncertain cause and pathogenesis, enigmatic epidemiology, and limited treatment options, the disease captured national attention and triggered considerable concern among parents of young children.

### Background.

AFM actually is a newly coined term for a subset of cases of the long-recognized syndrome of acute flaccid paralysis (AFP) (9, 10), in which cord myelitis is documented, typically by magnetic resonance imaging (MRI) visualization (3, 11). The term AFP subsumes additional causes of flaccid paralysis such as trauma, tumors, and immunopathologic disorders. Clinical descriptions of AFP appeared in medical textbooks in 1789. Clusters of cases began to be recognized in 1840, with larger epidemics documented in Sweden in 1881 and in the United States in 1894. Early, widespread epidemics came to be referred to as “poliomyelitis” (“polio” for short, derived from the Greek words for inflammation of the neural gray matter). In the late 1940s, the breakthrough (and Nobel Prize-winning) technology of viral cultivation in tissue culture led to the isolation of three infectious agents of epidemic polio (poliovirus types 1, 2, and 3), to further clinical and epidemiologic characterization of poliomyelitis, to effective polio vaccines, and to global polio eradication efforts, now in their final stages.

### Multiple broadly pathogenic nonpolio enteroviruses.

Poliovirus isolation techniques also led to discovery and characterization of a large, ubiquitous group of picornaviruses termed “enteroviruses,” containing not only the three polioviruses but also at least 110 “nonpolio enteroviruses” (NPEVs) (12). Unlike classical fecal-oral transmission of polioviruses, some NPEVs are more commonly transmitted by the respiratory route. NPEVs can cause a wide array of disease syndromes, including respiratory infections, conjunctivitis, myositis, pleurodynia, myocarditis, maculopapular and vesicular rashes, hand-foot-and-mouth disease, herpangina, meningitis, encephalitis, so-called “neonatal viral sepsis,” possibly type 1 diabetes, and—occasionally—sporadic AFM (9, 10). Indeed, the very first NPEV discovered (coxsackievirus A1, in 1947) was isolated from a child with AFM (13). In temperate climates, NPEVs circulate together endemically and epidemically every late summer/fall, causing localized outbreaks of aseptic meningitis and other conditions. Although immunity to NPEVs is near-universal by early childhood (10), infections continure to occur because there are many different NPEV types, some evolving rapidly (10).

Circulating NPEVs usually are replaced, in part or in whole, by other NPEVs in subsequent seasons (9, 10); however, some NPEVs may reappear at intervals of 2 or more years (8–10, 14, 15). For example, in Southeast Asia (but not in the rest of the world), EV-A71 has occurred in 2- to 3-year cycles (8); in previous decades, 5-year cycles were noted for EVA9 and EVB5; and various other cyclic patterns have been noted for different NPEVs (9, 14). The cycles presumably reflect factors such as viral transmissibility, population immunity, and possibly NPEV elicitation of cross-reactive immunity to shared epitopes. Short-interval cyclicity is consistent with viral hypertransmissibility, which leads to high population herd immunity that prevents further spread until such time as new annual birth cohorts of susceptible persons can sufficiently dilute it. Such patterns were well described for measles and other childhood diseases in the prevaccine era.

### NPEVs, AFM, and AFP.

Sporadic AFM inevitably appears at low incidence during widespread seasonal circulation of almost any NPEV. Some NPEVs, e.g., EV-A70 and EV-A71, have been historically more frequent causes of AFM than have others (8–10). Beginning in 1988, polio eradication efforts further clarified NPEV epidemiology via the global establishment of national surveillance systems to identify all cases of AFP. As many as 60,000 documented cases of nonpolio AFP are reported annually (16); cases are predominantly associated with NPEVs or Guillain-Barré syndrome, the latter of which is itself often associated with NPEVs (17, 18). Thus, multiple NPEVs have been closely linked to thousands of AFP and AFM cases for more than 3 decades.

### The global emergence of epidemic AFM.

Although sporadic AFM is not rare, its sudden appearance in epidemic form is unprecedented. Beginning in the summer and fall of 2012, California and other locales began to detect small, unexpected upticks in AFM cases featuring influenza-like respiratory prodromes and associated with various NPEVs. These AFM-associated NPEVs prominently included EV-D68 (19, 20), an historically obscure NPEV that had been reemerging globally since 2008 to cause pandemic respiratory disease (16, 21). The reemergence of EV-D68 was itself unprecedented. Although acute hemorrhagic conjunctivitis associated with EVA24v and with EV-D70 had caused global tropical air hub-to-air hub spread (9, 22), and hand-foot-and-mouth disease-associated EV-A71 had in recent decades caused Southeast Asian regional epidemics (8), no NPEV previously had been documented to reemerge from viral obscurity to spread pandemically.

By 2014 to 2015, large AFM epidemics began to appear across the United States and globally (Fig. 1); again, such outbreaks typically occurred in temporal association with EV-D68 epidemics (3, 4, 5, 19, 20). The EV-D68/AFM epidemiological association has since become unmistakable. Two unprecedented epidemics have been recurring in the same places at the same times: beginning in 2014, AFM epidemics in the United States have recurred in 2-year cycles of increasing magnitude, usually during seasonal EV-D68 circulation (Fig. 1). However, despite coclustering of AFM and EV-D68, viruses are often not identified from AFM cases and are almost never isolated from the cerebrospinal fluid (CSF). Confusion and doubt about the causes of AFM mounted in 2018.

### A hit-and-run infection?

There is an obvious paradox in temporal-geographic association between AFM and EV-D68, on the one hand, and difficulty detecting EV-D68 in AFM cases, on the other. In this regard, a precipitating EV-D68 infection, often with low-level viral replication (20), may well have run its course by the time of onset and diagnosis of AFM, several days to a week or more later. Early transient viremia during the respiratory prodrome might also have resolved by the time of AFM onset. Alternatively, local virus may cross the blood-brain barrier to extend proximally up nerve axons to the cord; this is believed to be the mechanism of ipsilateral trauma-associated “provocation poliomyelitis” (23). In addition, some NPEVs that spread by the respiratory route, including EV-D68, have low gastrointestinal tropism, hindering stool isolation (the standard poliovirus diagnostic technique).

It is noteworthy that while enterotropic polioviruses, and some other NPEVs that cause AFM, often can be isolated from stool for weeks, they, too, like nonenterotropic EV-D68, are uncommonly isolated from the CSF of paralytic cases (24). Similarly to epidemic polio, the AFM epidemic has been associated with cases of cranial nerve paralysis, bulbar paralysis, and meningoencephalitis (3, 20). Once viral damage to gray matter has occurred—via viral cytopathicity or a pathogenic immune response—intracellular virus may not be released into the (anatomically distant) spinal fluid and thus detected by lumbar puncture. Furthermore, although easily visible on MRI (3, 11), involved cord and bulbar gray matter cannot safely be biopsied to allow for direct virus isolation. For these reasons, EV-D68-induced pathogenic processes associated with early brief low-level viral replication and early transient viremia, or with direct axonal extension to internal cord gray matter, might well lead to AFM without providing good opportunity for viral detection.

Although EV-D68 appears to be good at covering its tracks, the epidemiologic evidence that EV-D68 is a major cause of epidemic AFM, while circumstantial, is nonetheless strong. Since historically many or even most cases of nonpolio AFP/AFM have been caused by circulating NPEVs (18, 24, 25), it is logical to suspect that during explosive EV-D68 epidemicity, many or most AFM cases would be caused by EV-D68 as well, even as AFM cases associated with other NPEVs continue to occur at lower background rates.

### Unanticipated plot twists.

As it unfolds, the AFM story seems to be getting more complicated. Preliminary U.S. data show that not only was epidemic AFM associated with EV-D68 in 2018, but also with EV-A71 (26), a well-known cause of both hand-foot-and-mouth disease and AFM that has been problematic in other regions of the world, but historically less so in the United States. Could we be entering some kind of new epidemic era, in which fundamental but unappreciated determinants of enterovirus evolution and spread are changing? In this context, it is also appropriate to consider whether epidemic AFM results only from high-level epidemic circulation of viruses such as EV-D68 and EV-A71 or, as some data suggest, from rapid viral evolution via mutation and recombination that leads to increased viral pathogenicity (20, 21, 24, 27–32). This is an extremely important question, and additional evidence will be crucial.

A related question is how to explain the paradox of EV-D68 epidemics in populations with virtually 100% preexisting neutralizing antibody to EV-D68, e.g., 2012–2013 preepidemic data from Kansas City, Missouri (33). Medical record reviews might identify missed or misclassified prior cases. More likely, perhaps, is that complex cross-reactive and cross-protective immunity of circulating enteroviruses drives viral evolution. It is of note that large epidemics of EV-A71 have in the past been associated with clade and subclade replacement (8). Furthermore, as is observed with other human viruses adapted to superficial mucosal infection, e.g., respiratory syncytial virus (RSV), noroviruses, and many others, the correlates of protective immunity to viruses such as EV-D68 may include mucosal immune factors such as IgA and local tissue-resident immune cells.

### Clinical conundrums.

As important as determining the cause of AFM is the establishment of optimal interventions to prevent, limit, or reverse neurologic damage that is often advanced at the time of clinical presentation. Attempts at treatment with interventions such as intravenous immunoglobulin, glucocorticoids, plasma exchange, and antiviral drugs such as pleconaril have been largely unsuccessful (34). Experimental nerve transfer to adjacent unaffected segments of the cord may offer improvement to some patients (35). Of greater promise is mounting evidence that early intensive physical therapy (36, 37), the value of which has been well documented for polio (37), may benefit nonpolio AFM patients as well. Therapy for polio was developed to be aggressive and comprehensive in addressing each of the three conceptual stages of acute, convalescent, and chronic polio paralytic disease (36, 37). Specific therapeutic goals targeted loss of flexibility, loss of muscle power, decreased vital capacity, potential for residual deformity, loss of skill, and loss of functional stamina. In recent decades, recognition of the postpolio syndrome, usually developing decades after initial paralysis, and with potential future implications for AFM patients, has led to additional physical therapy approaches (38). Physicians should consider physical therapy consultation at the earliest possible time after AFM onset.

### Research challenges.

A major challenge in the study of this disease is that AFM is an uncommon, sporadically occurring complication of a common infection that is difficult to diagnose with viral specificity. Moreover, one cannot predict where or when it will strike next, and the site of devastating tissue damage is inaccessible to direct study. Research progress is greatly impeded by lack of understanding of the natural history and pathogenesis of AFM disease, including viral pathogenesis, and by lack of understanding of AFM epidemiology, including aspects of cross-reactive immunity associated with the many and rapidly evolving NPEVs, especially EV-D68 and EV-A71. Perhaps, as has long been predicted, there is a “poliovirus niche” into which one or more NPEVs will evolve as we approach the eradication of polio (24, 39). It is conceivable that we may be seeing the early stages of such an evolution.

Until such time as the causes of the AFM epidemic are better understood, development of preventive vaccines will remain challenging. NPEV virus-specific diagnostics are needed, as are virus-specific serologic tests to support epidemiologic studies. Also needed are experimental animal models to study viral neurovirulence and neuroinvasiveness properties (32), genetic markers, and drug therapies (40). These and other research challenges are daunting because the NPEVs have been neglected for decades. Watching healthy children become permanently paralyzed virtually overnight by a seemingly random, lightning-strike disease is as heartbreaking today as it was in the polio era. The trajectory of AFM over the past 5 years suggests that the problem is getting worse, and so it is critical that we galvanize our efforts to learn more about, and respond adequately to, this ubiquitous, often crippling, continually reemerging group of viruses.
